# IgG from Adult Atopic Dermatitis (AD) Patients Induces Thymic IL-22 Production and CLA Expression on CD4+ T Cells: Possible Epigenetic Implications Mediated by miRNA

**DOI:** 10.3390/ijms23126867

**Published:** 2022-06-20

**Authors:** Thamires Rodrigues de Sousa, Beatriz Oliveira Fagundes, Andrezza Nascimento, Lorena Abreu Fernandes, Fábio da Ressureição Sgnotto, Raquel Leão Orfali, Valéria Aoki, Alberto José da Silva Duarte, Sabri Saeed Sanabani, Jefferson Russo Victor

**Affiliations:** 1 Laboratory of Medical Investigation LIM-56, Division of Dermatology, Medical School, University of Sao Paulo, Av. Dr. Enéas Carvalho de Aguiar 500, Sao Paulo 05403-000, Brazil; sousarthamires@gmail.com (T.R.d.S.); beatrizfagundesc@gmail.com (B.O.F.); raquelleao@hotmail.com (R.L.O.); valeria.aoki@gmail.com (V.A.); adjsduar@usp.br (A.J.d.S.D.); 2 Post-Graduation Program in Translational Medicine, Federal University of São Paulo, Sao Paulo 04039-002, Brazil; andrezza.ns@gmail.com (A.N.); lorena.abreu.fernandes@gmail.com (L.A.F.); 3 Division of Hematology, Medical School, University of Sao Paulo, Sao Paulo 01246-000, Brazil; fabio.house@hotmail.com; 4 Division of Pathology, Medical School, University of Sao Paulo, Sao Paulo 05403-000, Brazil; 5 Laboratory of Medical Investigation LIM-03, Division of Pathology, Medical School, University of Sao Paulo, Sao Paulo 05403-000, Brazil; 6 Faculdades Metropolitanas Unidas (FMU), Health Sciences School, Sao Paulo 04505-002, Brazil; 7 Medical School, Universidade Santo Amaro (UNISA), Sao Paulo 04829-300, Brazil

**Keywords:** human, thymus, Th22, IL-22, atopic dermatitis, miRNA

## Abstract

Atopic dermatitis (AD) is a common relapsing inflammatory skin disorder characterized by immune-mediated inflammation and epidermal barrier dysfunction. The pathogenesis of AD is multifactorial and has not been fully elucidated to date. This study aimed to evaluate whether serum IgG from adult AD patients could modulate the thymic maturation of IL-22-producing T cells and CLA+ T cells of non-atopic infants. Given that miRNAs regulate immune response genes, we evaluated whether miRNA expression is also altered in cultured thymocytes. Thymocytes were cultured with purified IgG from AD patients or control conditions (mock, Intravenous-IgG (IVIg), non-atopic IgG, or atopic non-AD IgG). Using flow cytometry analysis, we assessed the expression of CLA and intracellular levels of IL-4, IFN-γ, and IL-22 on double-positive T cells (DP T), CD4 T cells, or CD8 T cells. We also investigated the frequency of IgG isotypes and their direct interaction with the thymic T cells membrane. The miRNA profiles were evaluated by the Illumina small RNA-seq approach. MiRNA target gene prediction and enrichment analyses were performed using bioinformatics. Increased frequencies of IL-22 and CLA+ producing CD4+ T cells cultured with IgG of AD patients was seen in non-atopic infant thymocytes compared to all control conditions. No alterations were observed in the frequency of IgG isotypes among evaluated IgG pools. Evidence for a direct interaction between IgG and thymic DP T, CD4 T, and CD8 T cells is presented. The small RNA-seq analysis identified ten mature miRNAs that were modulated by AD IgG compared to mock condition (miR-181b-5p, hsa-miR-130b-3p, hsa-miR-26a-5p, hsa-miR-4497, has-miR-146a, hsa-let-7i-5p, hsa-miR-342-3p, has-miR-148a-3p, has-miR-92a and has-miR-4492). The prediction of the targetome of the seven dysregulated miRNAs between AD and mock control revealed 122 putative targets, and functional and pathway enrichment analyses were performed. Our results enhance our understanding of the mechanism by which IgG can collaborate in thymic T cells in the setting of infant AD.

## 1. Introduction

Atopic dermatitis (AD) is a common skin disease characterized by immune dysregulation, microbial dysbiosis, and epidermal barrier defects [1,2,3]. The world prevalence of AD in adults ranges from 2.1% to 4.9% [4]. Previous studies have shown that adults with AD had increased frequencies of Th22 cells within the skin-homing T-cell population (CLA+) than controls in adults patients compared to children, suggesting that age-dependent factors may modulate the “spreading” of Th22 [5,6]. The relevance of IL-22 in AD pathogenesis was evidenced in phase 2 clinical trials using a neutralizing anti-IL-22 monoclonal antibody (Fezakinumab), which resulted in progressive and sustained clinical improvements in adult patients with moderate or severe AD [7].

The debate over the mechanisms implicated in the modulatory effect of IgG on the thymic maturation of T cells has intensified in the last years [8,9,10], but there is still scarce evidence to explain the molecular basis of this process/hypothesis. Several studies have reported that different IgG antibody repertoires can play a pivotal role in mediating thymic and peripheral lymphocytes in murine and humans [11,12,13,14,15,16,17,18,19]. In AD, it has been reported that IgG from AD patients could favor the production of IL-17 and IL-10 by neonatal thymic CD4 and CD8 T cells and induce the expression of IL-4, IL-17, and IL-10 by neonatal thymic iNKT cells [20,21]. Such features corroborate with the phenotype of adult AD patients and explain the immune-mediated mechanism operative in AD pathogenesis [22]. The thymic maturation of other cell populations, including IL-22-producing CD4 T cells (Th22), is well established and plays a pivotal role in AD skin lesions [23]. 

Despite this, there is still a lot of work to be performed in order to understand the molecular pathways by which IgG impacts thymic T cells, and miRNA expression analysis has the potential to make a substantial contribution in this endeavor. miRNAs are a large group of short, non-coding RNAs that modulate the expression of several target genes by binding with imperfect complementarity to the 3′UTR of their messenger RNAs (mRNAs), causing mRNA degradation or inhibiting mRNA translation [24,25]. Previous studies have shown the modulation of miRNAs in the T-cells activation and cellular processes that are critical for the immune response [26,27], providing a possible path to elucidate the described regulatory effect of IgG molecules on thymic T cells, including Th22 cells. Given that the development of Th22 cells and their phenotypic characteristics, including the expression of homing molecules, may occur in the thymus, we asked whether IgG from adults with AD could modulate the non-atopic infant intra-thymic Th22 cells to be preferentially matured and acquire skin-homing phenotype (CLA+). Moreover, we aimed to perform a pilot mechanistic approach to assess potential implications for IgG isotypes frequencies, direct interaction of IgG thymic T cells membrane and expression of miRNA, which can mediate the observed effects. 

## 2. Patients and Methods

### 2.1. Patient Samples

Thymic tissues were obtained from 12 neonate patients younger than seven days of age (3.4 ± 0.54 days) who underwent corrective cardiac surgery at the Hospital do Coração (HCor), São Paulo, Brazil. Patients were required to meet the following criteria: no immunodeficiency, no genetic syndromes or allergic reactions, and no immunosuppressants. We evaluated the parental history of allergic disease, and only children of non-atopic parents were included in this study.

Similar to previous studies performed by our group [20,21], we recruited 14 adult patients diagnosed with AD according to Hanifin and Rajka and that were clinically categorized into moderate and/or severe AD using the eczema area and severity index (EASI) [28]. Eight of these patients were male, and six were female. The patients’ ages ranged from 24 to 35 years. The average disease duration was 26 years. The selected patients were admitted to the study during a scheduled visit to the Dermatology Outpatient Clinic service at the University of Sao Paulo, Brazil. None of the patients were given systemic corticosteroids (intravenous, oral, or potent topical) or immunosuppressants for at least four weeks. 

The control group comprised clinically diagnosed non-atopic (nAT, n = 23) and atopic patients without AD (AT, n = 26) aged 20 to 40 years. The inclusion criteria for the nAT control were negative IgE and skin prick test (SPT) for any tested allergens. The inclusion criteria for the AT subgroup were positive IgE and SPT for at least two tested allergens. The exclusion criteria for controls were AD symptoms, severe eczema, dermographism, and the use of any drug potentially affecting the SPT results. Whole blood samples were collected from all participants, and sera were fractionated, pooled, and stored at −80 °C.

Each thymus was obtained from a different donor, and a minimum of six independent experiments were performed to obtain the results. The ethics committees at the HCor and the School of Medicine at USP approved this study (CAAE: 15507613.4.0000.0060).

### 2.2. SPT

The SPTs were performed to classify nAT and AT individuals (control groups) following the European standards [29] with an adapted panel of allergens that included the profile of Brazilian allergens (i.e., *Dermatophagoides farinae*, *Dermatophagoides pteronyssimus*, *Aspergillus fulmigatus*, *Penicillium notatum*, *Alternaria alternata*, *Cladosporium herbarum*, *Blatella germânica*, *Periplaneta americana*, Alder, Birch, Hazel, Cultivated rye, Chicken, Canary Bird, Cat, and Dog). Briefly and as previously described [11], one drop of each allergen extract, histamine (positive control), or the allergen diluent (negative control) provided by IPI ASAC was applied to the volar forearm. A superficial skin puncture was made through each allergen or control drop using a hypodermic needle (Alko, Sao Paulo, Brazil) without inducing bleeding. After 15 min, the results were recorded by measuring the transverse diameter of each wheel reaction. Wheels with a diameter of 3 mm greater than the negative control were considered positive results.

### 2.3. Serum Anti-Allergen IgE Determination

The serum-specific IgE antibodies were evaluated to classify the nAT and AT individuals (control groups) following the manufacturers‘ instructions. Serum-specific IgE antibodies were measured with a multiplex immunoblot assay (EUROLINE Inhalation 2—EUROIMMUN AG, Lubek, Germany) using a method previously described by our group [30,31]. The tested extracts were: *Dermatophagoide farinae*, *Dermatophagoides pteronyssimus*, Cat, Dog, Horse, Guinea pig, rabbit, hamster, *Aspergillus fulmigatus*, *Penicillium notatum*, *Alternaria alternata*, *Cladosporium herbarum*, Timothy Grass, Cultivated rye, Alder, Birch, Hazel, Mugwort, and English plantain.

Briefly, the strips were incubated with patients’ sera; then, they were washed and incubated with alkaline phosphatase-conjugated goat anti-human IgG antibody. After the second washing step, the strips were incubated with the chromogen/substrate solution. The reaction was stopped by washing, and the strips were evaluated with the EUROLineScan software (EUROIMMUN, Lubek, Germany) to obtain semi-quantitative results.

The intensity of the bands was measured and converted into a score from zero to six and divided into the following concentrations (all expressed in kU/L): class 0 < 0.35; class 1 < 0.7; class 2 < 3.5; class 3 < 17.5; class 4 < 50.0; class 5 < 100.0; class 6 ≥ 100.0. According to the manufacturer’s instructions, individuals with a reactivity equal to or greater than class 2 frequently develop clinical symptoms; therefore, reactivity was considered in this study to include participants with reactivity equal to or above class 2.

### 2.4. Thymic Tissue Dissociation, Cell Isolation, and Storage

As previously described, thymocytes were released from the tissue samples using enzymatic dissociation [16]. As previously standardized by our group [21], the thymus was divided into small fragments and transferred to conical centrifuge tubes containing RPMI medium, 0.5 mg/mL collagenase A and 0.02 mg/mL DNase I (Roche Diagnostics, Mannheim, Germany). The digested fragments were homogenized, filtered through a plastic sieve to remove aggregates, and washed with the resulting cell suspensions. Next, the cells were resuspended, and the low-density fraction was collected via Ficoll gradient centrifugation (GE Healthcare Bio-Science, Uppsala, Sweden). The thymic cells were snap-frozen and stored in liquid nitrogen until use.

### 2.5. IgG Purification, Isotypes Evaluation, and Labeling

As previously described, IgG purification from pooled sera was performed using the Melon Gel IgG Spin Purification Kit (Thermo, Waltham, MA, USA) as previously described [14,19,21,32,33]. The purification gel was transferred to a column coupled to a polypropylene conical tube and was then centrifuged. The supernatant was discarded, and the purification gel was resuspended in a mild purification buffer at physiological pH. The supernatant was discarded, and the purification gel was again suspended in the same purification buffer. A pooled serum sample from each group (nAT, AT, or AD) was separately added to the gel, and the mixture was homogenized. The supernatant (purified IgG) was collected, sterilized, and stored at −80 °C for subsequent cell culture experiments. According to the manufacturer’s instructions, IgG concentration was determined using Coomassie Protein Assay Reagent (Pierce, WA, USA). The purity of IgG, evaluated by SDS-PAGE, was above 95%. Thus, this method is more effective than using protein A, but we cannot exclude that other antibody isotypes were potentially present as contaminants at low or undetectable concentrations. IgG isotypes (IgG1, IgG2, IgG3, and IgG4) were determined in purified IgG samples by ELISA (IgG Subclass Human ELISA Kit, ThermoFisher, Waltham, MA, USA), as per the manufacturer’s instructions.

We used the Zenon Human IgG Labeling Kit (Invitrogen, Waltham, MA, USA) following the manufacturer’s instructions for IgG labeling. Briefly, Alexa-647 fluorophore attached to the monovalent affinity-purified Fab fragments directly recognizes the Fc portion of human IgG. Because this labeling method is immunoselective, it excludes the staining of any other proteins, including non-IgG antibodies, resulting in specific IgG staining. Thymocytes were incubated for 30 min with labeled IgG or, as controls, with Zenon labeling and blocking reagents without purified IgG or unlabeled IgG only. To standardize this method, we confirmed that the previous incubation with the respective unlabeled purified IgG for each tested IgG pool could block the staining provided by labeled purified IgG.

### 2.6. Cell Culture and Flow Cytometry

Similar to previous studies from our group [20,21], the cell cultures were performed using purified IgG from adult AD patients and, as controls, we used commercially used intravenous IgG obtained from thousands of healthy donors (IVIg—Endobulin Kiovig, Baxter, Lessines, Belgium), purified IgG from non-atopic individuals (nAT), and purified IgG from atopic individuals (AT). Briefly, we individually evaluated thymocyte viability using a Neubauer chamber under an optical microscope (Laboroptik, Friedrichsdorf, Germany), and 1 × 10^6^ viable thymocytes were distributed to each well of a 48-well culture plate (CoStar, Glendale, CA, USA) with RPMI medium and 10% FCS (HyClone-III, Logan, UT, USA) with a total volume of 400µL, and they were cultured in the absence (Mock control condition) or the presence of 100 μg/mL of IgG purified from each group of IgG donors (AD, IVIg, nAT or AT). All thymocytes cultures were performed in individual experiments. The culture plate was incubated for six days (a standardized period for observation of lymphocyte maturation), and thymocytes were transferred to test tubes to perform extracellular (phenotypic) staining. Thymocytes were fixed with formaldehyde and stained with mouse anti-human CD4-V605, CD8-APC-Cy7, anti-CLA-PE, or isotype control antibodies to identify populations of immature double-positive T cells (CD4+CD8+; DP T cells), mature CD8 T cells (CD4-CD8+), mature CD4 T cells (CD4+CD8-), and the expression of CLA on each population.

To perform the intracellular detection of cytokines, thymocytes were separately cultured in the same conditions (mock control conditions or each group of IgG donors), but Brefeldin A (Sigma, Rehovot, Israel) was added to each well of the culture plate 12 h before cell staining [30,31]. This protocol was standardized using positive (phorbol 12-myristate 13-acetate—PMA) and negative controls (mock condition), and due to the absence of polyclonal stimulation, we could maintain Brefeldin A for 12 h without decreasing cell viability [22,32,34]. The culture conditions differ from phenotypic evaluation because Brefeldin A could impair the detection of non-constitutive surface molecules, including CLA, during the standardization period.

After the extracellular staining of CD4 and CD8 molecules, the samples were incubated with saponin. Afterward, the supernatant was discharged, and cells stained with mouse anti-human IFN-γ-V450, IL-4-APC, IL-22A-Alexa700, or isotype control conjugated with the corresponding fluorochromes (BD Pharmingen, New Jersey, NJ, USA).

For the cell viability analysis, extracellular staining was performed as described above, and the cells were stained with a Live/Dead (PE-Texas red) fluorescent reagent (ThermoFisher, Waltham, MA, USA). All antibodies, including labeled IgG, were titrated to define 1 μg as an optimal concentration for specific staining. Cell gating was determined using the isotype control values or the fluorochrome minus 1 (FMO) setting to all parameters. All extracellular and intracellular analyses were performed using viable cells.

Using an LSRII Fortessa flow cytometer (BD Biosciences, New Jersey, NJ, USA), 500,000 events per sample were acquired in the quadrant of lymphocytes. Compensation was performed using adsorbed microspheres (CompBeads-BD Biosciences, New Jersey, NJ, USA). Data analysis was performed using FlowJo software (Tree Star, New Jersey, NJ, USA).

### 2.7. RNA Extraction

An equal number of viable thymocytes from 20 neonate patients were pooled together to have sufficient cells and create a single sample for each experimental condition. RNA was extracted from cultured thymocytes from each condition with the miRCURY RNA Isolation kit (Exqon, Vedbæk, Denmark) as described in the manufacturer’s guidelines. The resulting RNA was eluted with RNase-free water and stored at −80 °C until use. sRNAs quantity was measured using a Qubit 2.0 fluorometer (ThermoFisher, Waltham, MA, USA). According to the manufacturer’s instructions, the total and sRNA concentration was initially measured using a Qubit 2.0 fluorometer with the RNA HS assay kit and microRNA Assay Kit (Thermo Fisher Scientific, Inc., Waltham, MA, USA).

### 2.8. sRNA Construction and Sequencing

Sequence libraries were generated using the TruSeq Small R.N.A. sample preparation kit (Illumina, San Diego, CA, USA) per the manufacturer’s instructions and a previously published protocol [31]. A total library pool of 4 nM was prepared using a MiSeq Reagent Kit v3 150 cycle followed by sequencing on a MiSeq system (Illumina, San Diego, CA, USA). The libraries were sequenced on a 150-SE run on the MiSeq with a 36 base single-end protocol [32]. After trimming adapter sequences and sequence quality testing, each library’s raw data were aligned to the human reference genome (hg19); then, they were combined to an expression matrix and processed with the Strand NGS version 3.1 (Strand Life Science, Bangalore, India). Only miRNAs with more than ten copies were considered for subsequent analysis. miRNAs with a fold-change ≥2 were supposed to be differentially expressed. All sequence data described here are available in the online Zenodo repository (https://doi.org/10.5281/zenodo.6470816; accessed on 14 June 2022).

### 2.9. Prediction, Gene Set Enrichment, and Functional Analysis of Target mRNA

We used the online platform miRWalk v3 [33] to predict the target gene of miRNAs. The target genes were obtained by the intersection of three prediction software programs: TargetScan, miRDB, and miRTarBase, based on the mirWALK v3 database. Functional and gene set enrichment analyses were carried out in miRWalk using the KEGG (KEGG Pathway Database, 2021) and REACTOME (Home-Reactome Pathway Database, 2021) databases to demonstrate the specific biological processes (BPs), cellular components (CCs), molecular functions (MFs) and pathways associated with the selected miRNAs of the resulting gene sets. Some of these pathways with *p*-values < 0.05 were selected for visualization.

## 3. Murine Methods

Detailed murine methods are included in the Supplementary Materials (Supplementary Methods).

## 4. Statistical Analysis

Statistical analysis was performed with GraphPad Prism 5.0 (GraphPad Software Inc., La Jolla, CA, USA). Data from in vitro studies were taken from 10 to 12 separate experiments with different thymus donors, as indicated in the following figures. Differences were considered significant at *p* ≤ 0.05, as assessed by one-way ANOVA (Kruskal–Wallis test, comparisons among three or more groups).

## 5. Results

### 5.1. IgG from Adult AD Patients Induces CLA Expression and IL-22 Production by Infant Non-Atopic Intra-Thymic CD4 T Cells with Similar Implications on Murine Cells

**Figure 1:**
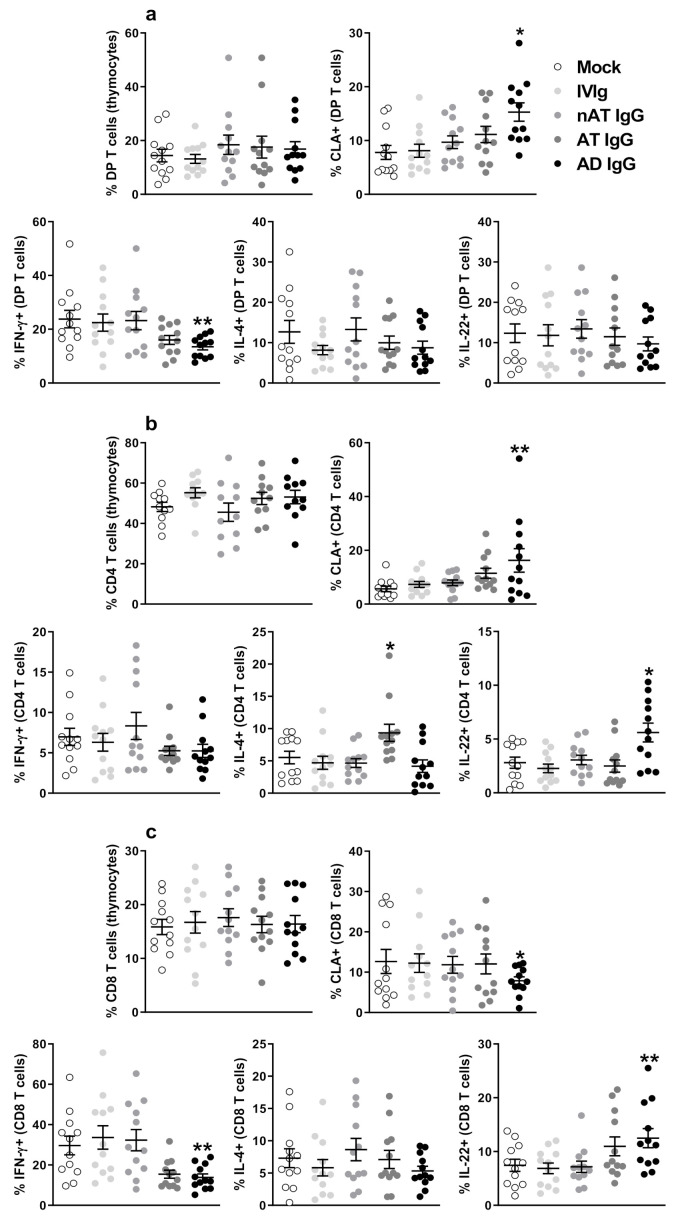
**Effect of purified adult AD IgG on infant non-atopic intra-thymic T cells.** Thymocytes from children under seven days old (n = 12) were evaluated after six days in culture in RPMI medium supplemented with FCS in the absence (mock) or presence of 100 µg/mL of commercially used purified IgG (IVIg), IgG purified from non-atopic individuals (nAT), IgG purified from atopic individuals (AT) or IgG purified from adult AD patients (AD). The frequencies of DP T, CD4 T, and CD8 T cells were evaluated (**a**), and the expression of CLA (**b**) or intracellular IFN-γ, IL-4, and IL-22 (**c**) were evaluated in these populations by flow cytometry. Each dot represents the value obtained from a different thymus. Bold lines represent the mean ± standard error. * *p* ≤ 0.05 compared to all other conditions; ** *p* ≤ 0.05 when compared to Mock. IVIg and nAT conditions. The absence of markings indicates that there was no statistical difference between the evaluated groups (*p* > 0.05).

The gating strategy to identify DP T, CD4 T, and CD8 T cells in the neonatal thymus is illustrated in Figure S1, and to identify CLA on DP T, CD4 T, and CD8 T cells, is shown in Figure S2. In our hands, the culture conditions did not influence the frequency of these populations (Figure 1a–c). The addition of AD IgG to DP T and CD4 T cells induced a significant increase in CLA and CD4 T cells expression compared to all control conditions (mock, IVIg, nAT, and AT—Figure 1b). In contrast, the addition of AD IgG induced a significant suppression of CLA on CD8 T cells (Figure 1c).

Next, we determined whether AD IgG had effects on the production of IFN-γ, IL-4, and IL-22 by DP, CD4, and CD8 T cells from neonatal thymic tissue. The gating strategy employed to identify intracellular cytokines is shown in Figures S3–S5. On DP T cells, only AD IgG reduces IFN-γ levels without alterations in IL-4 and IL-22 (Figure 1a). The addition of AD IgG into cultured CD4 T cells significantly increased the expression of IL-22 compared to mock and all control IgG conditions (IVIg, nAT, and AT—Figure 1b). Again, the addition of AD IgG to CD8 T cells induced a significant elevation in the expression of IL-22 compared to mock, IVIg, and nAT conditions (Figure 1c). Interestingly, IL-4 was increased in response to AT IgG addition to cultured CD4 T cells (Figure 1b). Because of the novelty of these findings, we conducted a pilot study in which we adapted our in vitro protocol to murine thymocytes. To that end, we produced five different immune serums and cultured neonatal (3-day-old) mouse thymocytes in the presence of purified IgG from each immune serum at the same concentration and time as human thymocytes to evaluate if any modulatory effect could be observed. Is this pilot study, the intracellular production of the main dicotomic cytokines in murine T cells, IFN-γ (Th1 profile) and IL-4 (Th2 profile) was investigated. Our findings indicate that pure IgG from different immune serum modulates the frequency of IFN-γ or IL-4 in murine DP T, CD4 T and CD8T cells in a distinct manner. Detailed methodology and results are available in the Supplementary Materials (Figure S6 and Supplementary Methods).

### 5.2. IgG Isotypes Are Not Related to the Modulatory Effect and the Possible Involvement of Direct Interactions with Maturing T Cells

**Figure 2:**
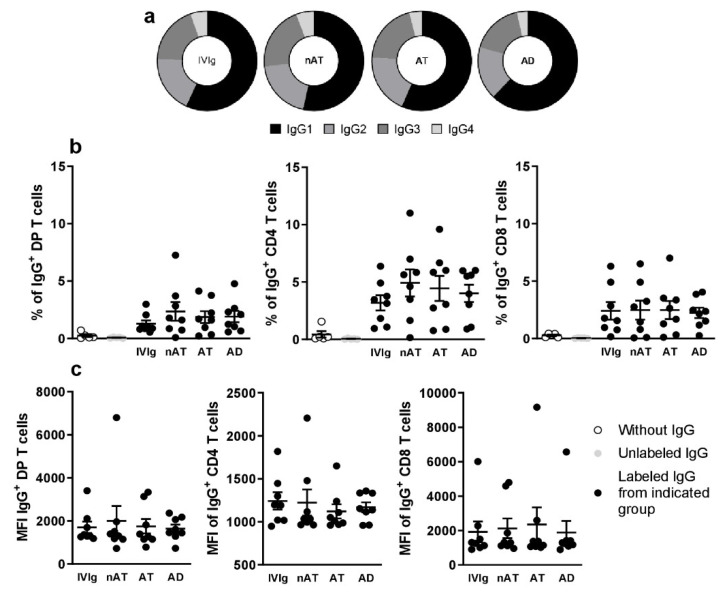
**Evaluation of IgG isotypes frequency and the direct interaction with thymic T cells.** The frequency of IgG1, IgG2, IgG3, and IgG4 isotypes was evaluated in all purified IgG pools (**a**). Thymocytes from children under seven days old (n = 8) were incubated for 30 min with labeling kit reagents (without IgG), unlabeled IgG, labeled IVIg, labeled nAT IgG, labeled AT IgG, or labeled AD IgG. The frequency (**b**) and intensity (**c**) of IgG staining (IgG+) on thymic T cells were demonstrated. Pie charts represent the frequency of each IgG isotype within the total amount of detected IgG. Each dot represents the value obtained from a different thymus. Bold lines represent the mean ± standard error. The absence of markings indicates that there was no statistical difference between the evaluated groups (*p* > 0.05).

In a brief and pilot mechanistic approach, we evaluated if the observed effects in response to IgG could be related to IgG isotypes (IgG1, IgG2, IgG3, and IgG4). As shown in Figure 2a, the frequencies of the IgG isotypes were similar between all tested purified IgG. We also assessed if purified IgG could directly interact with DP, CD4, and CD8 T cells (Gating strategy illustrated in Figure S7), and we noticed that all tested IgG could be detected bound to DP T, CD4 T, and CD8 T cells membranes at similar frequencies (Figure 2b). After evaluating the membrane-complexed IgG intensity, we found no difference among purified IgGs (Figure 2c).

### 5.3. Identification of Differentially Expressed miRNAs and Their Target Genes

The analysis of small RNA-seq was limited to miRNAs due to the fact that previous studies focused mostly on miRNA expression and the fact that they play key regulatory roles in various biological processes and diseases.

**Figure 3:**
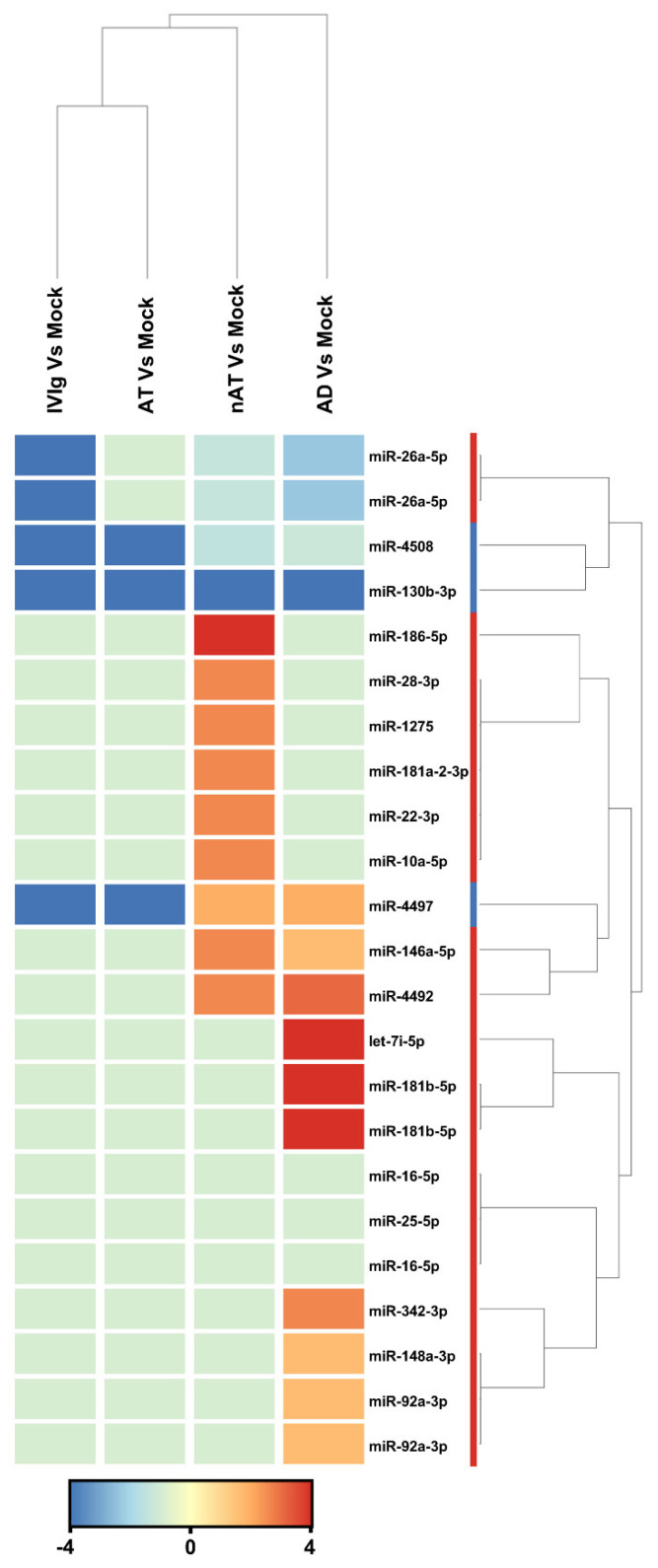
**miRNAs that were modulated in response to each IgG compared to mock condition**. Thymocytes from children under seven days old (n = 20) were evaluated after six days in culture in RPMI medium supplemented with FCS in the absence (mock) or presence of 100 µg/mL of commercially used purified IgG (IVIg), IgG purified from non-atopic individuals (nAT), IgG purified from atopic individuals (AT) or IgG purified from adult AD patients (AD). Unsupervised hierarchical clustering demonstrating 20 differentially expressed mature hsa-miRNAs between each IgG and the mock condition. The has-miRs clustering tree is displayed to the right, forming two major clusters. The color scale at the bottom indicates the fold change expression levels of mature hsa-miRs across in both samples: red color indicates upregulation and blue downmodulation.

According to this study, there were a total of 2104 active miRNAs identified, and of those, 23 were found to be differentially expressed among the four groups (AD, NAT, AT-D, and IVIg) compared to the mock control (fold change > 2), as demonstrated in Figure 3. Among these miRNAs, the study found seven non-redundant miRNAs that could distinguish between AD and mock control (fold change > 2). A total of five miRNAs (hsa-miR-181b-5p, hsa-miR-4492, hsa-let-7i-5p, hsa-miR-4497, and hsa-miR-342-3p) were upregulated, while two miRNAs (hsa-miR-26a-5p and hsa-miR-130b-3p) were downregulated. Based on the differentially expressed miRNAs, cluster analysis successfully separated the AD from mock control (Figure 3). hsa-miR-181b-5p and hsa-let-7i-5p (fold change 6.17 and 4.67, respectively) were the most upregulated miRNAs, and hsa-miR-130b-3p (fold change −4.5) was the most downregulated. The deep sequencing approach also identified 12 miRNAs that were upregulated in AT-nr thymocytes and 11 that were downregulated when compared to a mock control. Finally, all 23 miRNA were downregulated in AT-D and IVIg thymocytes relative to the mock control.

**Figure 4:**
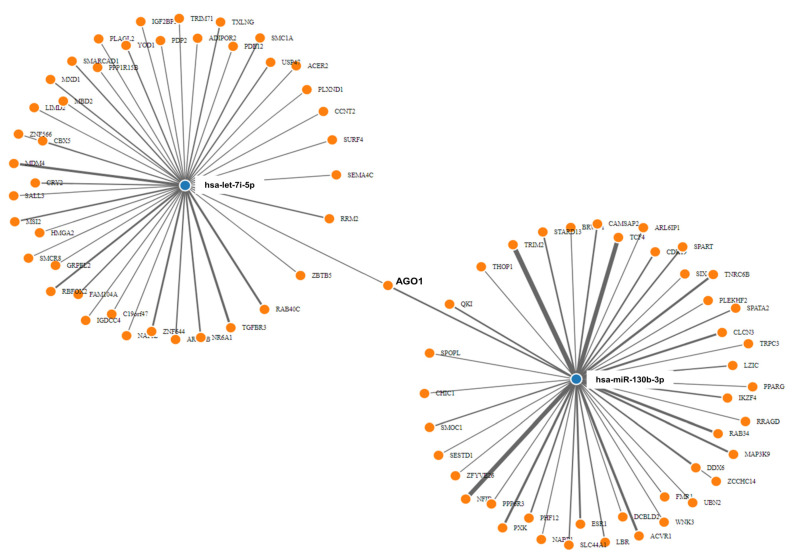
**miRNAs–target genes interaction**. Interaction network between hsa-let-7i and hsa-mir-130b deregulated mature miRNAs and their target genes generated by MirWalk v3. Blue circles represent miRNAs, while orange circles represent mRNAs. The more connections between miRNAs and genes, the more links within the network.

The prediction of the targetome of the seven dysregulated miRNAs between AD and Mock control revealed 122 putative targets from miRWalk. Hsa-let-7i-5p and hsa-miR-130b-3p were the miRNAs with the highest number (over 40) of experimentally validated targets. Next, GSEA was conducted on predicted targets per miRNA to reveal the biological processes related to each miRNA. The list of these pathways according to their P-values is presented in Table 1. Inspection of the network (Figure 4) revealed that hsa-let-7i-5p and hsa-miR-130b-3p synergistically target AGO1. The AGO1 is one of the Argonaut proteins that has been shown to reside in intracellular structures known as Processing-bodies (P-bodies). This discrete area of the cells is believed to govern the cellular mRNA turnover [33].

**Table 1:** Reactome pathways with adjusted *p* value ≤ 0.05.

Name	Hits	Pop Hits	List Total	Pop Total	Genes	*p*-Value	Adjusted *p*-Value (BH)
R-HSA-381340_Transcriptional regulation of white adipocyte differentiation	7	133	69	10812	CDK8;CDK19;TBL1XR1;PPARG	0.0	0.0
R-HSA-8986944_Transcriptional Regulation by MECP2	8	74	69	10812	TNRC6B;TRPC3;AGO1;TBL1XR1;PPARG;LBR	0.0	0.0
R-HSA-73857_RNA Polymerase II Transcription	27	1628	69	10812	ESR1;TNRC6B;NABP1;RRM2;CBX5;KRBOX4;TRPC3;ZNF566;ZNF268;MDM4;CCNT2;CDK8;NR6A1;PHC3;AGO1;TBL1XR1;CPSF2;PPARG;LBR;RRAGD	0.0001	0.0008
R-HSA-74160_Gene expression (Transcription)	30	1838	69	10812	ESR1;TNRC6B;NABP1;RRM2;TET2;CBX5;KRBOX4;TRPC3;ZNF566;ZNF268;NFIB;MDM4;CCNT2;CDK8;NR6A1;PHC3;AGO1;TBL1XR1;CPSF2;PPARG;LBR;MBD2;RRAGD	0.0001	0.0008
R-HSA-212436_Generic Transcription Pathway	23	1439	69	10812	ESR1;TNRC6B;RRM2;CBX5;KRBOX4;TRPC3;ZNF566;ZNF268;MDM4;CCNT2;CDK8;NR6A1;PHC3;AGO1;TBL1XR1;PPARG;LBR;RRAGD	0.0003	0.0015
R-HSA-6807070_PTEN Regulation	7	177	69	10812	TNRC6B;PHC3;AGO1;PPARG;RRAGD	0.0003	0.0015
R-HSA-8943724_Regulation of PTEN gene transcription	5	98	69	10812	PHC3;PPARG;RRAGD	0.0006	0.0026
R-HSA-1257604_PIP3 activates AKT signaling	8	307	69	10812	ESR1;TNRC6B;PHC3;AGO1;PPARG;RRAGD	0.0014	0.0052
R-HSA-1989781_PPARA activates gene expression	5	134	69	10812	CDK8;CDK19;TBL1XR1;PPARG	0.0024	0.0075
R-HSA-400206_Regulation of lipid metabolism by PPARalpha	5	136	69	10812	CDK8;CDK19;TBL1XR1;PPARG	0.0025	0.0075
R-HSA-9006925_Intracellular signaling by second messengers	8	360	69	10812	ESR1;TNRC6B;PHC3;AGO1;PPARG;RRAGD	0.0036	0.0098
R-HSA-3108232_SUMO E3 ligases SUMOylate target proteins	6	231	69	10812	ESR1;CBX5;SMC1A;PHC3;PPARG	0.0052	0.013
R-HSA-2990846_SUMOylation	6	239	69	10812	ESR1;CBX5;SMC1A;PHC3;PPARG	0.0061	0.0141
R-HSA-2559583_Cellular Senescence	5	208	69	10812	TNRC6B;MDM4;HMGA2;PHC3;AGO1	0.0137	0.0294
R-HSA-9006931_Signaling by Nuclear Receptors	6	305	69	10812	ESR1;TNRC6B;SMC1A;AGO1;TBL1XR1;SCD	0.0178	0.0356
R-HSA-556833_Metabolism of lipids	11	786	69	10812	OSBPL8;ACER2;CDK8;SLC44A1;CDK19;TBL1XR1;PPARG;LBR;PLPP3;SCD	0.0195	0.0366

### 5.4. Functional and Pathway Enrichment Analyses

GO annotation and KEGG pathway enrichment analysis of all 122 target genes were performed by the miRWalk v3.0 online tool. The results of the GO BPs analysis revealed that the target genes of dysregulated miRNAs mainly were enriched in the transforming growth factor-beta receptor signaling pathway (GO:0007179), negative regulation of translation (GO:0017148), and DNA-templated transcription (GO:0045892) (Table S1). In the GO CC category, the target genes of the dysregulated miRNAs were significantly enriched in the cytoplasm and nucleus (GO:0000932, GO:0005844, and GO:0000118). In the MF category, the target genes were significantly enriched in mRNA binding (GO:0003729), nuclear receptor activity (GO:0004879), transcriptional activator activity (GO:0003714), SMAD binding (GO:0046332), and DNA binding (GO:0003677). In addition, KEGG pathway analysis revealed that the target genes of the seven dysregulated miRNAs were uniquely enriched in the pathway of the transcriptional misregulation in cancer (hsa05202) (adjusted *p*-value = 0.01).

## 6. Discussion

Although AD is one of the most common diseases, many aspects of its pathogenesis remain incompletely understood. It has already been demonstrated that adults and infants with AD differ in immune polarization [5]. These differences include the differential modulation of CD4 T cells cytokine profiles, including Th1, Th17, and Th22 [5,35]. Comparing the pathological development of AD between infants and adults is technically complex, and therefore, the identification of biological factors that can explain this age-related development is of great importance.

Hence, observing human and murine studies that correlate the modulatory effect of IgG according to its repertoire in allergies [16,17,19,20,33,34], HIV-1 infection [13], and AD [20,21], we hypothesized that IgG from AD patients could be elected as a biological-inducing factor naturally developed by individuals prone to develop AD. To test this hypothesis, we used a standardized protocol of human thymic culture with IgG to investigate the effect of adults’ AD IgG repertoire on non-atopic infant thymus, focusing on the production of a Th22 profile and expression of the skin-homing molecule CLA [5,24].

We used IVIg, a pool of purified human IgG antibodies obtained from thousands of healthy individuals [36], and IgG obtained from non-atopic or atopic individuals (without AD) with the same purification method used to obtain purified AD patients’ IgG. These conditions control the potential influence of the purification process, contamination by other biologically active molecules, or even other non-determined factors related to atopy development but non-related to AD.

Our results indicated that at the immature stage of T cells maturation (DP T cells), IgG from AD individuals could induce the overexpression of CLA, which was maintained on mature CD4 T cells but not on CD8 T cells, where an opposite effect was revealed. CLA is the primary skin-homing molecule expressed by T cells and is considered a peripheral biomarker of AD [37]. Therefore, its substantial overexpression on DP and CD4 T cells suggests that an IgG repertoire prone to developing AD can modulate intra-thymic T cell maturation favoring that CD4 T cells migrate to the skin, which was not observed in response to healthy or atopic (non-AD) IgG donors.

The evaluation of IL-22-producing T cells in this study reveals that CD4 T cells respond to the addition of AD IgG by acquiring an intense Th22 spectrum without the modulation of Th1 and Th2 profiles. Several studies have reported different modulatory effects on AD CD4 T cells, including endophenotypic variations related to age, race, and ethnicity [38]. However, our current study’s pronounced effect on Th22 seems to be specifically mediated by AD IgG, since we used several IgG controls and, among them, the atopic IgG (derived from Th2-related individuals) could induce a diverse effect with a Th2 to augment compared to all other conditions. The effect of atopic IgG inducing a Th2 profile corroborates with a similar study demonstrating a Th1 inhibition on thymic T cells mediated by atopic IgG [31]. Additionally, we performed a brief murine approach that could indicate the ability of murine IgG to modulate Th1 and Th2 profiles in neonatal thymocytes, but we did not pursue this further, because it was not the study’s objective. However, additional mechanistic approaches are need to fully understand these observations.

Based on these translational results, we hypothesize that IgG-based immunoregulatory therapies would effectively treat AD. The latest evidence in this field has demonstrated that the intramuscular administration of total autologous IgG provides clinical improvements and a systemic immunomodulatory effect in adolescent and adult patients with moderate to severe AD [39,40]. Thus, this treatment may represent a cost-efficient and safe therapeutic approach in patients with AD, although the underlying mechanism is not yet fully known [41].

Aiming to elucidate possible IgG-mediated therapeutic effects, we also performed a pilot mechanistic approach. Since direct evidence of the interactions between IgG and human thymic immature cells is absent in the literature, we aimed to investigate this approach. We first demonstrated that our results are not due to IgG isotypes, since the frequency of IgG1, IgG2, IgG3, and IgG4 were similar among all used purified IgG pools. This observation is mainly important, since elevated levels of IgG4 isotype are related to atopic and allergic disorders [42,43], although differential IgG4 patterns may be observed among different allergic diseases [44].

Next, we evaluated if purified IgG could directly interact with thymic T cells. Our findings revealed the detection of all labeled purified IgG pools on the membrane of immature DP T cells, mature CD4 T, and mature CD8 T cells, although at relatively low levels. We also demonstrated that the intensity of labeled purified IgG detected on thymic T cells was similar among evaluated IgG pools. These results suggested that the observed effect does not elapse from a more frequent or intense interaction of IgG antibodies with the T cells membrane. Therefore, we suggest that the effect occurs because of differences in the pattern of molecules that each pool of purified IgG can recognize or interact with.

Since thymic DP, CD4, and CD8 T cells do not express IgG receptors, we hypothesize that the observed interactions may be idiotypically mediated and thus may involve several membrane molecules that we could not determine in the present study. Nevertheless, these observations are unprecedented in the literature and, unfortunately, they could not be confirmed by confocal microscopy because the labeling intensity of CD4 and CD8 molecules on thymic lymphocytes did not allow the efficient detection of labeled IgG, as our group already performed it in a similar approach with γδ T cells [45]. This limitation needs to be overcome in future studies.

The anti-idiotypic antibody induction of T cell immunity was first demonstrated in 1975 [46]. It was demonstrated in 2003 that anti-TCR antibodies exist in pooled purified human IgG. These antibodies are directed to recognize “public” idiotypes found in TCRβ chains exerting, thereby modulating the expression of these T cells [47]. Later, it was also experimentally proven that natural and autoantibodies against TCRβ can alter the Th1-mediated inflammatory response [48]. As reviewed in 2014, multiple studies focused on elucidating the immunomodulatory effects of the therapeutic use of IgG (intravenous IgG—IVIg) have already demonstrate the involvement of idiotypic interactions as mediators of the modulatory effects on T cells immunity [49].

The pattern of these idiotypic interactions mediated by IgG may be altered by naturally developed and environmentally-stimulated IgG repertoire, resulting in distinct IgG repertoires for every immunological background (i.e., non-atopic, atopic, or AD in our study). Consequently, these distinct sets of IgG may interact with their respective sets of receptors (including TCRs), inducing differential effects on patterns that have yet to be understood. A recently proposed hypothesis, named “the hooks without bait”, discussed the modulatory implications of idiotypic interactions on murine and human experimental observations and is closely related to our findings [50].

Complementing our approaches, we further investigate if the evidenced IgG/T cells’ direct interaction could modulate the expression of miRNAs. Although a few miRNAs have been identified as AD modulators, we sought to verify if our in vitro model could reveal a possible epigenetic effect mediated by AD IgG in the human thymus.

Although there was no evidence to assign the roles of IgG antibodies in the modulation of human thymic miRNAs, our results show that 17 miRNAs are differentially expressed in human thymocytes in response to the used IgG pools compared to the mock condition, in which ten mature miRNAs were modulated by AD IgG (miR-181b-5p, hsa-miR-130b-3p, hsa-miR-26a-5p, hsa-miR-4497, has-miR-146a, hsa-let-7i-5p, hsa-miR-342-3p, has-miR-148a-3p, has-miR-92a, and has-miR-4492).

The modulation of these miRNAs is consistent with previous studies and is involved in several cellular processes, including cell proliferation, differentiation, immune response, and apoptosis [51,52,53,54,55,56]. For instance, a high expression of miR-181b-5p levels has been detected in the thymus [27]. The gene ontology study analysis by Ghorbani et al. [57] revealed targets that exhibited an over-representation of immune pathways related to the T-cell receptor, corroborating our hypothesis that IgG may mediate the engagement of T-cell receptors. Regarding has-miR-181b-5p, its low expression was described in allergic asthma patients with high peripheral eosinophil frequency, which could be reverted after inhaled corticosteroid treatment [58]. This evidence suggested differential epigenetic regulation from these miRNAs between different atopic diseases. This issue needs to be better investigated.

In the context of AD, the study by Acevedo and colleagues [59] revealed the dysregulation of 16 miRNAs in skin homing CD4+CLA+ T cells from AD patients, including the upregulation of miR-181b-5p and miR-130b-3p in CD4+CLA+ T cells. Our results revealed an upregulation of miR-181b-5p but a downregulation of miR-130b-3p in the infant thymus cultured with AD IgG. One possible explanation for this difference might be the non-atopic genetic background of the thymus samples used.

Our results also demonstrated that IgG from AD patients could downregulate hsa-miR-26a-5p in human thymocytes. The downregulation of hsa-miR-26a-5p was described in skin lesions of AD patients in three different studies [60,61,62]. This miRNA possibly can regulate the hyaluronan synthase 3 (*HAS3*) gene involved in the hyaluronic acid synthesis. Malaisse and colleagues found that HAS3 expression was higher in AD lesions compared to healthy and nonlesional AD skin [63]. Additionally, has-miR-26a was reduced in human peripheral Th22 cells, corroborating with our cellular observations [64]. These findings suggested that IgG from AD patients could induce miRNA expression, which operates as a co-regulator of gene expression in thymocytes to regulate the immune response to AD skin lesions and Th22 induction.

Our results also demonstrated that AD IgG could induce the expression of hsa-miR-4497. Although less studied, hsa-miR-4497 induction has been observed in HaCaT cells (human keratinocytes) exposed to UVB radiation. In these cells, the hsa-miR-4497 expression could be related to the inhibition of cell proliferation, production of pro-inflammatory cytokines (TNF-a, IL-18, IL-6, and IL-1b), and induction of apoptosis. In this study, the downregulation of hsa-miR-4497 significantly inhibited the effects of UVB radiation in HaCaT cells, whereas the overexpression of miR-4497 further enhanced these effects [65]. This evidence suggested that the induction of hsa-miR-4497 may promote keratinocytes injury, which is a feature of patients with AD. Thymocytes cultured with AT IgG and IVIg had the opposite effect, indicating a difference in epigenetic regulation that needs to be investigated further.

Our results demonstrated that AD IgG could increase the expression of has-miR-146a. A previous study has shown that hsa-miR-146a is upregulated in AD chronic skin inflammation, where it exerts an anti-inflammatory role by the suppressing innate immune responses in keratinocytes [66]. Another study has found that has-miR-146a is highly expressed in CD4+ CD25high T cells (Treg cells), myeloid dendritic cells, and mast cells, corroborating the concept that it could play an important anti-inflammatory role during skin inflammation [67]. Interestingly, this same miRNA was induced by nAT IgG, suggesting that nAT IgG could be involved in the induction of anti-inflammatory epigenetic factors that will need to be explored further in future investigations.

The overexpression of has-let-7i-5p has previously been associated to non-allergic (fine particulate matter induced) asthmatic patients’ plasma, implying a link between its expression and an inflammatory state but with no mechanistic explanation [68]. The upregulation of hsa-miR-342-3p in leprosy skin lesions has been described [69], but there is no evidence that it is related to atopic diseases. Regarding has-miR-148a-3p, we found that this miRNA is highly expressed in human breast milk, especially in the first days of breastfeeding, but there is no direct evidence linking it to atopic diseases [70].

Finally, hsa-miR-92a has been found to be upregulated in the nasal mucosa of patients with allergic rhinitis (AR) and the plasma of patients with systemic lupus erythematosus (SLE) [71,72]. The latter studies suggested that hsa-miR-92a could be used as a biomarker for AR or SLE. However, our findings suggest that hsa-miR-92a may also play a role in the pathogenesis of AD disease. There were no previous references to hsa-miR-4492 in the literature.

The transcriptional regulation of white adipocyte differentiation and transcriptional regulation by MECP2 (methyl-CpG-binding protein 2) were the most crucial reactome enrichment pathways. It is worth noting that differentiating preadipocytes are a rich source of antimicrobial peptides (AMPs), and the production of these antibiotics is provoked by bacterial exposure [73]. Indeed, AD, is linked to patients’ chronic susceptibility to bacterial and viral infections [74], and it is caused by a lack of AMP production. MECP2 is a transcriptional regulator that controls the expression of methylation sensitive genes. Mutations in MECP2 have been reported in patients with autism [75]. MeCP2 is also implicated in systemic lupus erythematosus [76] and in a number of neuropsychiatric and neurological diseases, and its deregulation can have functional effects [77]. There is currently no known association between MeCP2 and AD, although our study suggests a link. Further studies are necessary to confirm the involvement of these pathways in the pathogenesis of AD.

GO enrichment analyses were performed for the miRNA target genes. The results showed that a lot of GO annotations were enriched. Particularly, the GO terms “cytoplasm, P-bodies; cytoplasmic ribonucleoprotein”, “nucleus”, and “mRNA_binding”, and “transforming growth factor beta receptor signaling pathway” were the most significantly enriched. An earlier study by Wu et al. [78] showed a spontaneous increase in telomerase, a ribonucleoprotein enzyme involved with cellular proliferation and cellular senescence, and activity in lymphocytes from patients with AD. Therefore, we hypothesized a correlation between the dysregulation of miRNAs and these GO terms.

In conclusion, we found that IgG from adult AD patients can stimulate the neonatal thymic maturation of Th22 and CLA+ CD4 T cells by an undescribed mechanism that possibly involves direct idiotypic interactions of IgG with thymic T cells, and that involves a complex epigenetic regulation that needs to be elucidated. Together, these observations may collaborate with the future elucidation of the modulatory effect of IgG on the pathophysiology of AD.

## Supplementary Materials

The following supporting information can be downloaded at: https://www.mdpi.com/article/10.3390/ijms23126867/s1.
