# Supplementary material for: IgG from Adult Atopic Dermatitis (AD) Patients Induces Thymic IL-22 Production and CLA Expression on CD4+ T Cells: Possible Epigenetic Implications Mediated by miRNA
Source: Int J Mol Sci. 2022 Jun 20;23(12):6867. doi: 10.3390/ijms23126867 (PMC9224968; doi:10.3390/ijms23126867)
Supplement: Supplementary file 1 [file ijms-23-06867-s001.zip › Supplementary figures S1 to S7 Minor1.pdf]

Supplemental Figures S1 to S7

Title: IgG from adult atopic dermatitis (AD) patients induces thymic IL-22 production and CLA expression on CD4+ T cells: possible epigenetic implications mediated by miRNA

Authors: Thamires Rodrigues de Sousa; Beatriz Oliveira Fagundes; Andrezza Nascimento; Lorena Abreu Fernandes; Fábio da Ressureição Sgnotto; Raquel Leão Orfali, MD, PhD; Valéria Aoki, MD, PhD; Alberto José da Silva Duarte, MD, PhD, Sabri Saeed Sanabani, PhD, and Jefferson Russo Victor, PhD.

Figure S1

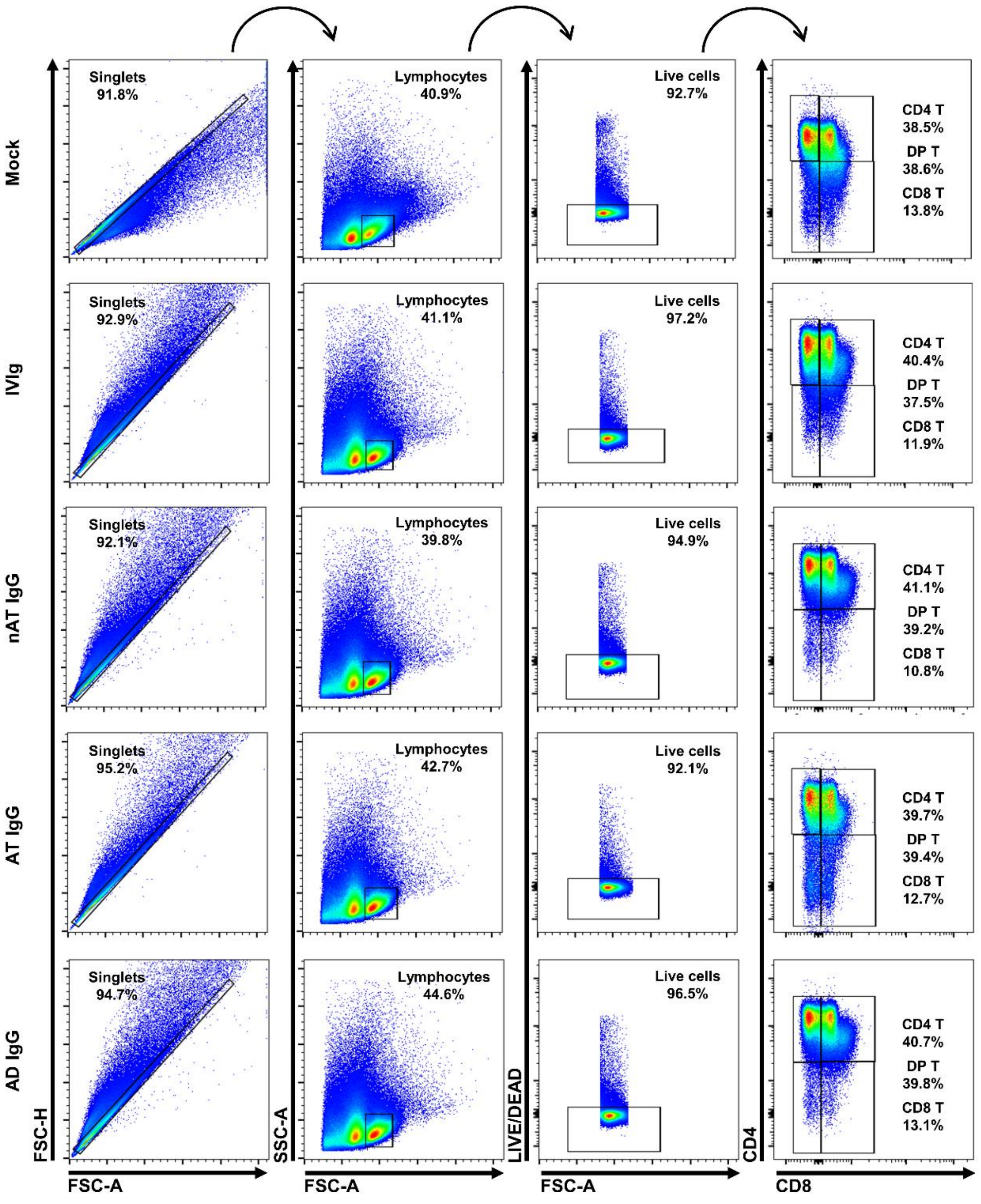

Figure S1: Illustrative dot plots of the gating strategy used to identify DP, CD4, and CD8 T cells. Each sample was acquired by a consecutive approach using a single-cell gate (determined by SSC-A/SSC-H parameters), a lymphocyte gate (determined by relative SSC-A/FSC-A), a Live cells gate, and gating to determine DP T (CD4+CD8+), CD4 T (CD4+CD8-) and CD8 T (CD4-CD8+) cells. This figure illustrates the gating strategy used to determine T cells on mock, IVIg, nAT IgG, AT IgG, and AD IgG culture conditions (from upper to lower panels).

Figure S2

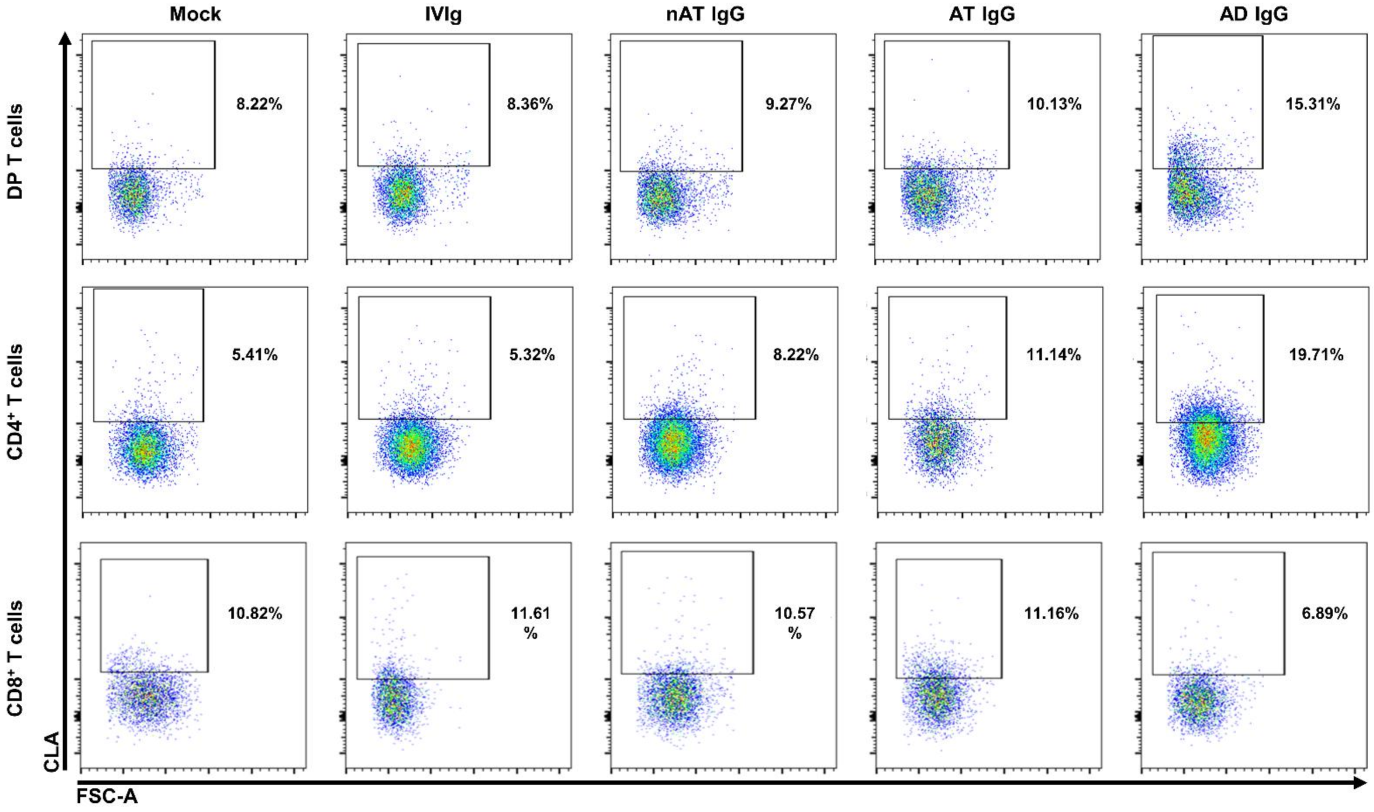

Figure S2: Illustrative dot plots of the gating strategy used to evaluate CLA expression on DP, CD4, and CD8 T cells after culture. Each sample was acquired by a consecutive approach using a single-cell gate (determined by SSC-A/SSC-H parameters), a lymphocyte gate (determined by relative SSC-A/FSC-A), a Live cells gate, and gating to determine DP T, $CD4^+$ T, and $CD8^+$ T cells. Samples were then acquired as CLA^+. This figure illustrates the gating strategy used to evaluate CLA expression on mock, IVIg, nAT IgG, ATIgG, and AD IgG culture conditions (from left to right panels).

Figure S3

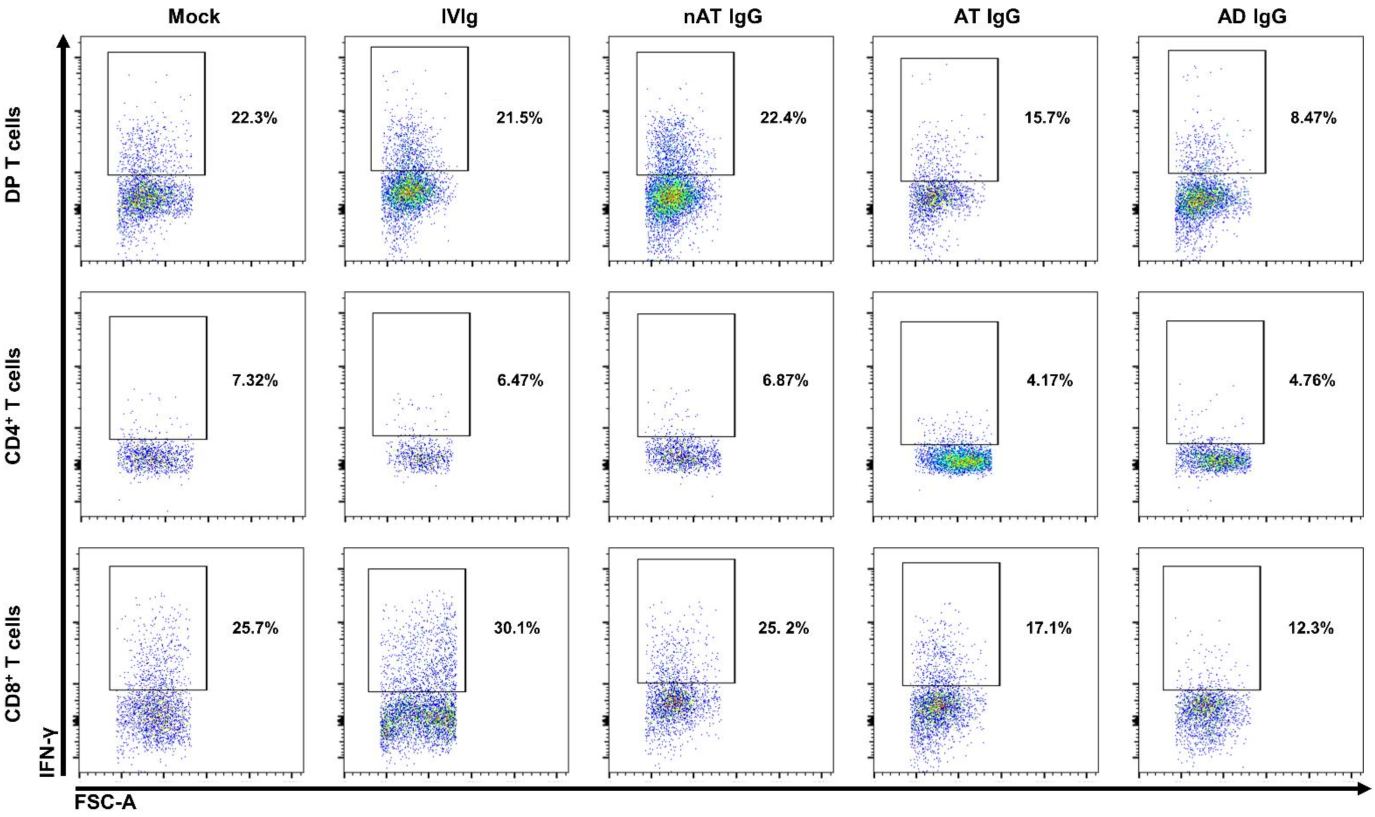

Figure S3: Illustrative dot plots of the gating strategy used to evaluate the production of IFN-γ on DP, CD4, and CD8 T cells after culture. Each sample was acquired by a consecutive approach using a single-cell gate (determined by SSC-A/SSC-H parameters), a lymphocyte gate (determined by relative SSC-A/FSC-A), a Live cells gate, and gating to determine DP T, $CD4^+$ T, and $CD8^+$ T cells. Samples were then acquired as IFN-γ^+. This figure illustrates the gating strategy used to evaluate IFN-γ expression on mock, IVIg, nAT IgG, AT IgG, and AD IgG culture conditions (from left to right panels).

Figure S4

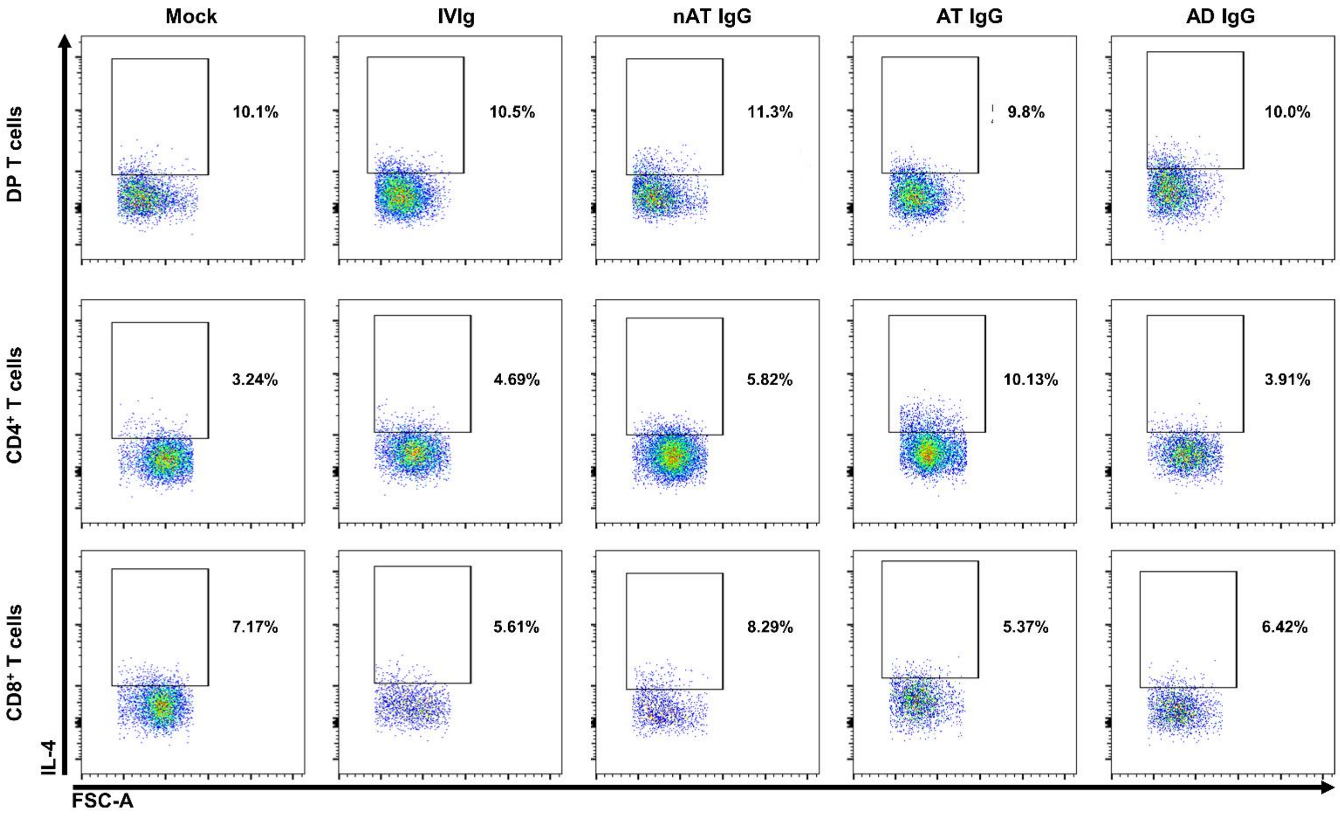

Figure S4: Illustrative dot plots of the gating strategy used to evaluate the production of IL-4 on DP, CD4, and CD8 T cells after culture. Each sample was acquired by a consecutive approach using a single-cell gate (determined by SSC-A/SSC-H parameters), a lymphocyte gate (determined by relative SSC-A/FSC-A), a Live cells gate, and gating to determine DP T, CD4$^+$ T, and CD8$^+$ T cells. Samples were then acquired as IL-4$^+$. This figure illustrates the gating strategy used to evaluate IL-4 production on mock, IVIg, nAT IgG, AT IgG, and AD IgG culture conditions (from left to right panels).

Figure S5

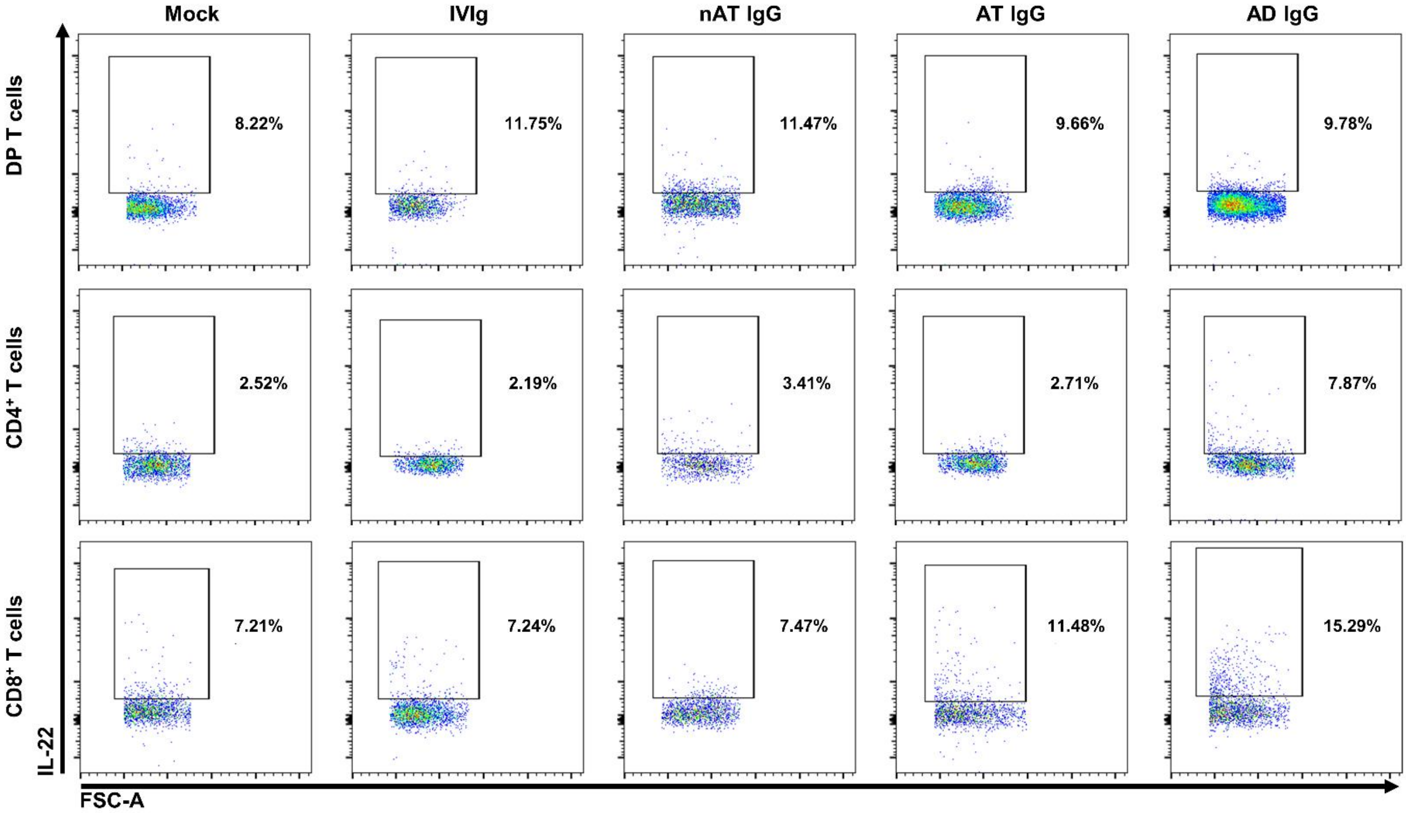

Figure S5: Illustrative dot plots of the gating strategy used to evaluate the production of IL-22 on DP, CD4, and CD8 T cells after culture. Each sample was acquired by a consecutive approach using a single-cell gate (determined by SSC-A/SSC-H parameters), a lymphocyte gate (determined by relative SSC-A/FSC-A), a Live cells gate, and gating to determine DP T, CD4^{+} T, and CD8^{+} T cells. Samples were then acquired as IL-22^{+}. This figure illustrates the gating strategy used to evaluate IL-22 production on mock, IVIg, nAT IgG, At IgG, and AD IgG culture conditions (from left to right panels).

Figure S6

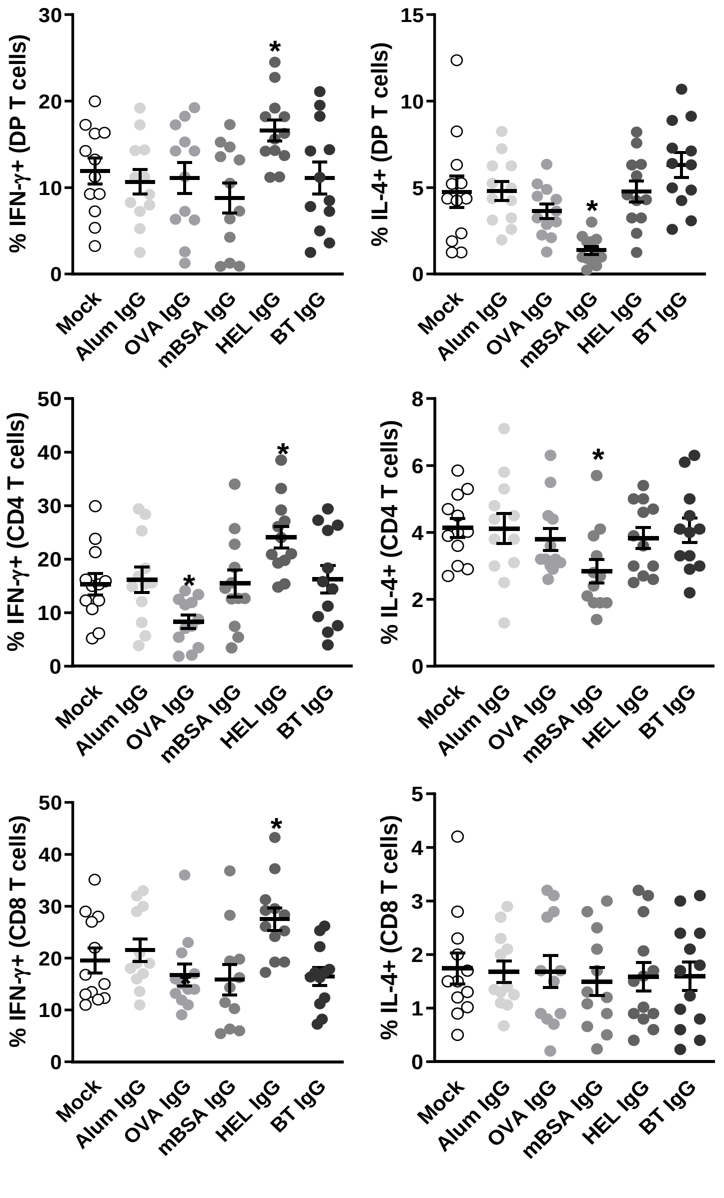

Figure S6: Pilot approach evaluating the modulatory effect of purified murine IgG on the modulation of neonatal murine thymic T cells. Thymocytes from neonatal mice (3 days old, n=12) were evaluated after six days in culture in RPMI medium supplemented with FCS in the absence (Mock) or presence of 100 µg/mL of IgG purified from Alum- (Alum IgG), OVA- (OVA IgG), mBSA- (mBSA IgG), HEL- (HEL IgG) or BT-immunized (BT IgG) adult female mice. The frequencies of DP T, CD4 T, and CD8 T cells were evaluated (a), and the expression intracellular IFN-γ and IL-4 were evaluated in these populations by flow cytometry. Each dot represents the value obtained from a different murine thymus. Bold lines represent the mean ± standard error. *p≤0.05 compared to all control conditions (Mock and Alum IgG).

Figure S7

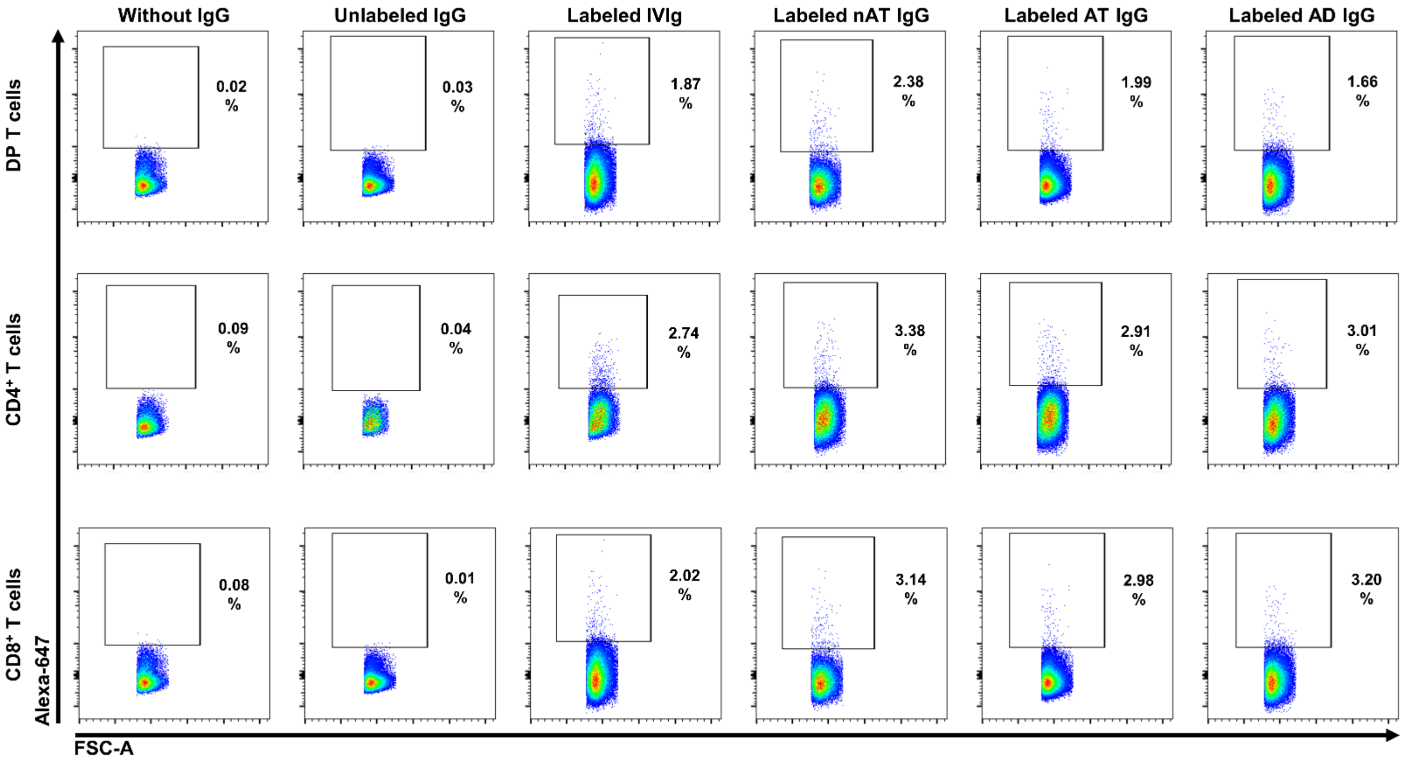

Figure S7: Illustrative dot plots of the gating strategy used to evaluate IgG-membrane interaction on DP, CD4, and CD8 T cells. Each sample was acquired by a consecutive approach using a single-cell gate (determined by SSC-A/SSC-H parameters), a lymphocyte gate (determined by relative SSC-A/FSC-A), a Live cells gate, and gating to determine DP T, CD4^{+} T, and CD8^{+} T cells. Samples were then acquired, from left to right, in the presence of Zenon human IgG labeling kit reagents but without IgG (Without IgG), in the presence of unlabeled IgG, in the presence of labeled IVIg, in the presence of labeled nAT IgG, in the presence of labeled AT IgG, or in the presence of labeled AD IgG. This figure illustrates the gating strategy used to detect IgG-membrane interactions in the cited conditions from left to right panels.
