# Supplementary material for: IgG from Adult Atopic Dermatitis (AD) Patients Induces Thymic IL-22 Production and CLA Expression on CD4+ T Cells: Possible Epigenetic Implications Mediated by miRNA
Source: Int J Mol Sci. 2022 Jun 20;23(12):6867. doi: 10.3390/ijms23126867 (PMC9224968; doi:10.3390/ijms23126867)
Supplement: Supplementary file 1 [file ijms-23-06867-s001.zip › Supplementary methods.pdf]

Supplementary Methods

Title: IgG from adult atopic dermatitis (AD) patients induces thymic IL-22 production and CLA expression on CD4+ T cells: possible epigenetic implications mediated by miRNA

Authors: Thamires Rodrigues de Sousa; Beatriz Oliveira Fagundes; Andrezza Nascimento; Lorena Abreu Fernandes; Fábio da Ressureição Sgnotto; Raquel Leão Orfali; Valéria Aoki; Alberto José da Silva Duarte, Sabri Saeed Sanabani, and Jefferson Russo Victor

Murine Immunization

C57BL/6 WT female adult mice were immunized subcutaneously with 6 mg of Alum (FURP, Sao Paulo, Brazil) alone or supplemented with 150 μg of Ovalbumin (OVA, Sigma-Aldrich), 150 μg of Methylated Bovine Serum Albumin (mBSA, Sigma-Aldrich), 150 μg of Hen Egg Lysozyme (HEL, Sigma-Aldrich), or 150 μg *Blomia tropicalis* extract (BT, **IPI**-ASAC Espanha). In saline, these animals were boosted intraperitoneally after 10 and 20 days with 150 μg of the same antigen used for priming. The females that were immunized with Alum only were boosted with saline alone. Blood samples were collected 14 days after the second booster.

Purification of Murine IgG

IgG was purified from pooled serum (For each antigen used or Alum alone) following the Melon Gel IgG Spin Purification Kit protocol (Thermo, Waltham, MA, USA). Purified IgG was collected, sterilized using 0.20-micron filters (Corning, Darmstadt, Germany), and stored at -80 °C for cell culture experiments. Following the manufacturer's instructions, IgG concentrations were determined using Coomassie Protein Assay Reagent (Pierce, Waltham, MA, USA). As evaluated by SDS-PAGE, the purity of IgG was greater than 95%. All pools were evaluated for IgA, IgM, and IgE antibodies, all of which were undetectable.

Thymus Cell Suspensions

Thymus was collected aseptically from 3 days old mice, and cells were isolated for culture or flow cytometry analysis. Single-cell suspensions were prepared with cell strainers (BD Biosciences, MA, USA) and placed in Petri dishes containing RPMI medium. The cell suspensions were treated with lysis buffer (Biosource—ACK Lysis Buffer, Rockville, MD, USA) for 2 min and then washed twice with RPMI medium. The cells were subsequently resuspended in 1 mL of RPMI medium with 10% FetalClone III (FC-III—HyClone, Logan, UT, USA), and cell viability was quantified with 0.5% trypan blue in a Neubauer chamber. All cell suspension analyses in this study were performed with individual samples.

Murine Cell Culture

To investigate the in vitro effect of purified maternal IgG on offspring T cells and thymocytes from 3-d.o. normal offspring were cultured for 120 h at a density of 3×10^6 cells/mL in RPMI 1640 (Gibco) supplemented with 10% FC-III (HyClone, Logan, UT, USA) in the mock condition or the presence of 100 μg/mL purified IgG from Alum-immunized, OVA-immunized, mBSA-immunized, HEL-immunized or BT-immunized mice. All culture wells were administered 10 mg/mL brefeldin A (Sigma-Aldrich) 24 h before flow cytometry analysis to evaluate intracellular cytokine production.

Murine Flow Cytometry

The evaluation of intracellular cytokine production was performed using a previously described method [829]. Single-cell suspensions were prepared in flow cytometry buffer (PBS, 1% BSA) for surface staining. Anti-CD4 and anti-CD8 antibodies directly conjugated to Cy-Chrome or APC (BD Biosciences) were used at their optimal concentrations, as determined by titration experiments and according to the origin tissue. For intracellular staining, the cells were first surface-stained, fixed (PBS, 1% formaldehyde—Sigma), permeabilized (PBS, 0.5% saponin—Sigma), and subjected to intracellular staining with anti-IFN-γ and anti-IL-4 directly conjugated to PE or FITC (BD Biosciences). Instrument compensation was performed using microbeads coated with anti-rat/anti-hamster antibodies (CompBeads, BD Biosciences) and their conjugated

antibodies. Acquisition of 300,000 events per sample was performed in the lymphocyte quadrant (as determined by ratio size/granularity) on an LSRFortessa cytometer (BD Biosciences, San Jose, CA, USA), and analysis was performed using FlowJo software 10.1 (Tree Star).
